# Rodent malaria-resistant strains of the mosquito, *Anopheles gambiae*, have slower population growth than -susceptible strains

**DOI:** 10.1186/1471-2148-9-76

**Published:** 2009-04-20

**Authors:** Maarten J Voordouw, Bradley R Anholt, Pam J Taylor, Hilary Hurd

**Affiliations:** 1 Department of Biology, University of Victoria, PO Box 3020, Station CSC, Victoria, British Columbia, V8W 3N5, Canada; 2 Centre for Applied Entomology and Parasitology, School of Life Sciences, Keele University, Staffordshire ST5 5BG, UK

## Abstract

**Background:**

Trade-offs between anti-parasite defence mechanisms and other life history traits limit the evolution of host resistance to parasites and have important implications for understanding diseases such as malaria. Mosquitoes have not evolved complete resistance to malaria parasites and one hypothesis is that anti-malaria defence mechanisms are costly.

**Results:**

We used matrix population models to compare the population growth rates among lines of *Anopheles gambiae *that had been selected for resistance or high susceptibility to the rodent malaria parasite, *Plasmodium yoelii nigeriensis*. The population growth rate of the resistant line was significantly lower than that of the highly susceptible and the unselected control lines, regardless of whether mosquitoes were infected with *Plasmodium *or not. The lower population growth of malaria-resistant mosquitoes was caused by reduced post blood-feeding survival of females and poor egg hatching.

**Conclusion:**

With respect to eradicating malaria, the strategy of releasing *Plasmodium*-resistant *Anopheles *mosquitoes is unlikely to be successful if the costs of *Plasmodium*-resistance in the field are as great as the ones measured in this study. High densities of malaria-resistant mosquitoes would have to be maintained by continuous release from captive breeding facilities.

## Background

Parasites exert strong selection on their hosts to evolve resistance mechanisms that avoid or reduce the negative fitness consequences of infection. Host resistance includes any mechanism (behaviour, morphology, physiological or immune response) that results in the avoidance, clearance, or tolerance of parasitic infections [1]. Theory predicts that the evolution of these resistance mechanisms in the host is constrained by antagonistic pleiotropy, when one allele affects two or more traits with opposite effects on fitness [2,3]. Such costs of evolving resistance have been demonstrated by measuring negative genetic correlations (genetic trade-offs) between immunity and other life history traits in selection or quantitative genetic experiments [4-9].

Most of these studies measure several life history traits and report that some of these fitness components are negatively correlated with host resistance whereas others are not [6,7]. Almost none of these studies have tried to combine multiple fitness components into a single measure of lifetime fitness. This is important because the evolution of immunity, like any other trait, ultimately depends on its correlation with lifetime fitness [10].

In organisms with age or stage-structured life histories, one widely recognized measure of lifetime fitness is the geometric population growth rate, λ [11]. To estimate λ one uses matrix algebra to model the life cycle of the organism [12]. The parameter λ depends on the age-specific or stage-specific vital rates, which are difficult to measure for a single individual. Hence, this approach works best when comparing groups of individuals (e.g., parasite-resistant versus parasite-susceptible host genotypes). One advantage of combining multiple life history traits into a single measure of lifetime fitness such as λ is that it avoids the problem of multiple comparisons and type I error. Another advantage is that all life history traits can be expressed in units of λ. This allows us to use parameter estimates (i.e., biological significance) rather than p-values (statistical significance) to determine which life history traits caused the difference in lifetime fitness. The purpose of the present study is to demonstrate the utility of this approach using a previously published data set by Hurd et al. [13] on the fitness costs and benefits of malaria resistance in *Anopheles gambiae *mosquitoes. *A. gambiae *is the most important vector of human malaria in Africa and is the main target of attempts to engineer a malaria-resistant mosquito [14]. We review the host-parasite interactions between the mosquito host and the malaria parasite and the study of Hurd et al. [13] below.

Malaria parasites (genus *Plasmodium*) are transmitted between vertebrate hosts during blood feeding by female *Anopheles *mosquitoes. Following an infected blood meal the sexual stages of *Plasmodium *release gametes into the mosquito mid-gut and fertilization occurs. The zygotes develop into ookinetes that cross the epithelial layer of the mosquito mid-gut within 24 hours of fertilization. The ookinetes transform into oocysts that grow for one to two weeks on the exterior of the mid-gut before releasing thousands of sporozoites into the mosquito hemocoel. The sporozoites migrate to the mosquito salivary glands where they are transmitted to the next vertebrate host during blood feeding.

From the perspective of the female mosquito, *Plasmodium *is a parasite because it reduces both reproductive success [15-17] and survival [18]. Mosquitoes have evolved a number of defence mechanisms to protect themselves from *Plasmodium *[19-22]. Natural populations of mosquitoes have considerable genetic variation in *Plasmodium *resistance [23]. Several reviews have speculated that this variation is maintained by genetic trade-offs between *Plasmodium *resistance and other life history traits [24,25]. Hurd et al. [13] tested this hypothesis in *A. gambiae *by comparing eight different life history traits between strains that they had selected for resistance or high susceptibility to the rodent malaria parasite, *P. yoelii nigeriensis*. They found no significant differences in the two life history traits they deemed most important, longevity and fertility and concluded there was no difference in fitness between the refractory and the highly susceptible genotypes.

We re-analyzed the data of Hurd et al. [13] using stage-classified matrix population models [12] to combine all of the life history traits into an estimate of λ. We found that the malaria-resistant *A. gambiae *mosquitoes have a lower population growth rate than malaria-susceptible mosquitoes (regardless of whether mosquitoes were infected with *Plasmodium*). Our estimates of λ suggest that the population size of the malaria-resistant line will be half that of the susceptible line in just 23 days. This is the first study to show that there are high fitness costs for mosquitoes to evolve resistance to malaria, which has important implications for strategies that seek to reduce malaria transmission by releasing malaria-resistant mosquitoes.

## Results

### Differences in λ between malaria-refractory and -susceptible genotypes

We used matrix population models to estimate the geometric population growth rate (λ) for genotypes of *A. gambiae *that were resistant or highly susceptible to the rodent malaria parasite *P. yoelii nigeriensis*, as well as an unselected control genotype (see Methods). The mean λ (± standard error) of the refractory genotype (1.062 ± 0.0113) was lower than that of the highly susceptible (1.097 ± 0.0092) and the unselected control genotype (1.100 ± 0.0135). For explaining the most variation in λ with the fewest possible parameters, the best model according to our model selection criteria (low AIC score, parsimony) included the main effects of group, environment, genotype and the group:environment interaction (model 5 in Table 1). In this model, genotype (F_2,16 _= 5.675, p = 0.014) and the group:environment interaction (F_4,16 _= 3.506, p = 0.031) were statistically significant and accounted for 21.7% and 26.8% of the variation in λ, respectively (Table 2). For model 5, the first planned comparison found that the difference between the mean λ of the unselected control genotype and that of the selected genotypes (i.e., the refractory and highly susceptible genotypes combined) was not significant (p = 0.073). This indicates that the selection regime did not affect λ (e.g. via inbreeding). The second planned comparison found that the mean λ of the refractory genotype was significantly lower than that of the highly susceptible genotype (p = 0.014). This indicates that the evolution of *Plasmodium *resistance in the refractory genotype reduced λ. The population doubling times of the refractory, the highly susceptible, and the unselected control genotypes are 11.5 days, 7.5 days, and 7.3 days, respectively. This means that after 23 days, a population of refractory genotypes will be half the size of a population of highly susceptible or unselected control genotypes (2 doublings versus 3).

**Table 1:** Linear models of the population growth rate (λ).

id	Model structure	param	res s.e. (*10^-3^)	r^2^	F	p	AIC
1	λ ~ D + E + G + D:E + D:G + E:G	18	27.58	0.455	2.208	0.127	-110.1
2	λ ~ D + E + G + D:E + D:G	14	25.86	0.521	3.022	0.031	-110.7
3	λ ~ D + E + G + D:E + E:G	14	27.63	0.453	2.539	0.057	-107.1
4	λ ~ D + E + G + D:G + E:G	14	36.30	0.056	1.111	0.432	-92.3
5	λ ~ D + E + G + D:E	10	26.34	0.503	3.633	0.011	-109.9
6	λ ~ D + E + G + D:G	10	33.31	0.206	1.672	0.173	-97.2
7	λ ~ D + E + G + E:G	10	34.36	0.155	1.475	0.236	-95.5
8	λ ~ D + E + G	6	32.27	0.254	2.476	0.059	-100.9
9	λ ~ D + E + D:E	8	32.47	0.245	2.055	0.098	-99.4
10	λ ~ D + G + D:G	8	33.95	0.175	1.688	0.170	-97.0
11	λ ~ E + G + E:G	8	36.13	0.065	1.226	0.339	-93.6
12	λ ~ D + E	4	36.12	0.066	1.456	0.249	-96.2
13	λ ~ D + G	4	32.90	0.225	2.883	0.046	-101.3
14	λ ~ E + G	4	34.00	0.172	2.349	0.086	-99.5
15	λ ~ D	2	36.34	0.054	1.745	0.196	-97.6
16	λ ~ E	2	37.26	0.006	1.077	0.356	-96.2
17	λ ~ G	2	34.42	0.152	3.324	0.053	-100.5
18	λ ~ 1	0	37.37				-97.9

λ was modeled as a linear function of 3 factors: group (black, red, green), environment (uninfected, infected, stressed), and genotype (unselected control, refractory, highly susceptible), and their interaction terms. For each of the 18 models, the model structure shows the factors and interaction terms included in the model. Each model was assigned a unique identification number (id). Also shown for each model are the number of parameters (param), residual standard error (res s.e.), the adjusted r^2 ^value (r^2^), the F-statistic of the model (F), the p-value of the model (p), and Akaike's Information Criterion (AIC). For each model, the residual degrees of freedom are 26 minus the number of parameters. In the model structure column, group, environment, and genotype are abbreviated as D, E, and G, respectively.

**Table 2:** Significance testing of the terms in the best model.

Term	df	SS (*10^-3^)	MS (*10^-3^)	F	p	r^2^
group	2	4.609	2.305	3.322	0.062	12.7
environment	2	2.991	1.495	2.156	0.148	8.2
genotype	2	7.874	3.937	5.675	0.014	21.7
group:environment	4	9.728	2.432	3.506	0.031	26.8
Residuals	16	11.100	0.694			30.6

Total	26	36.302				100.0

The ANOVA table is shown for model 2, which was the best model in Table 1 (i.e. the model within 1 unit of the lowest AIC score and with the fewest number of parameters). For each term in the model, the degrees of freedom (df), the sum of squares (SS), the mean square (MS), the F-statistic (F) the p-value (p), and the partial r^2 ^value (r^2^) are shown.

**Figure 1:**
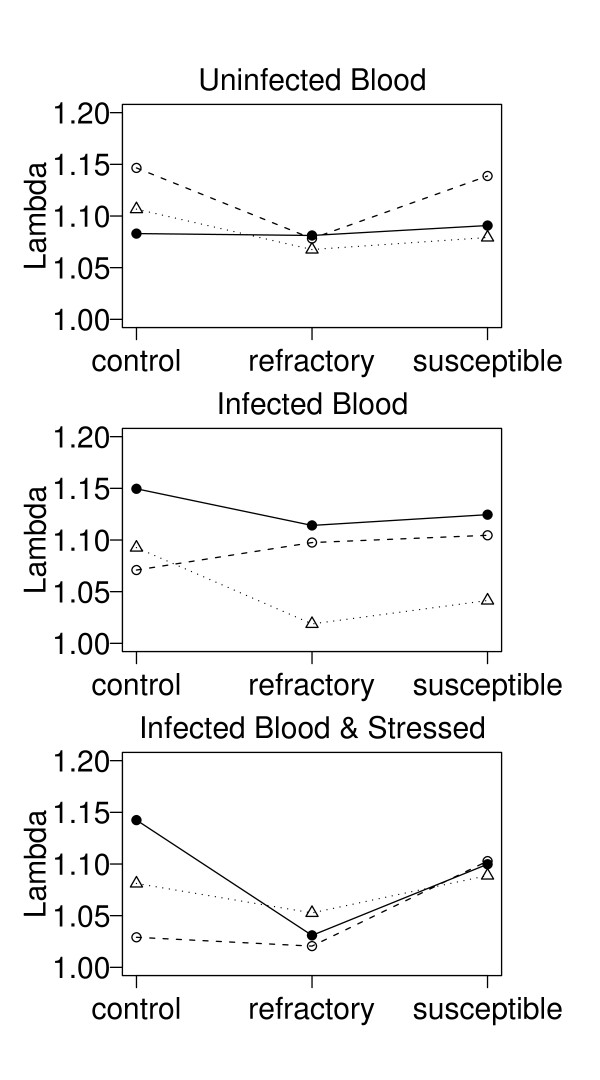
**The population growth rates of the 3 genotypes in 3 different environments**. The population growth rate (λ = Lambda) is shown as a function of group (black, red, green), environment (uninfected blood meal, infected blood meal, infected blood meal and subsequently stressed), and genotype (unselected control, refractory, highly susceptible). The groups are shown with different symbols and line types (black = solid circles and solid line; red = open circles and dashed line; green = open triangles and dotted line), the environments are shown in different panels, and the genotypes are shown on the x-axis. Each of the lines that connect three points in Figure 2 represents a different mouse.

The significant group:environment interaction (Table 2) suggests that the effect of group is contingent on the environment (and vice versa). For example, for the three environments – uninfected blood, infected blood, infected blood and stressed – females from the red group have the highest, intermediate, and lowest population growth (averaged across genotypes), respectively (Figure 1). Alternatively, because each of the 9 combinations of group and environment were fed on a different mouse, random variation among mice may be causing the group:environment interaction. Our best model (model 5 in Table 1) is therefore identical to one that would fit genotype and mouse as fixed factors with no genotype:mouse interaction.

### Differences in the life cycle parameters between genotypes

The refractory genotype was outperformed by the highly susceptible genotype on all the life cycle parameters except pupation success (Table 3). The refractory genotype was also outperformed by the unselected control genotype on all the life cycle parameters except egg production (Table 3). The unselected control genotype had higher pupation and egg laying success than the highly susceptible genotype (Table 3). After correcting for multiple comparisons, α = 0.05/21 = 0.002, none of differences in Table 3 are statistically significant.

**Table 3:** Differences in the life cycle parameters among genotypes.

	control – susceptible		control – refractory		susceptible – refractory	
Parameter	mean	p	mean	p	mean	p
p.hatch	-0.003	0.968	0.116	0.032	0.119	0.035
p.pupate	0.137	0.025	0.079	0.026	-0.058	0.257
p.blood	-0.025	0.535	0.059	0.203	0.084	0.014
p.surv.blood	-0.013	0.236	0.106	0.083	0.119	0.044
p.surv.ovip	-0.038	0.274	0.021	0.624	0.059	0.072
eggs.tot	-8.564	0.076	-6.016	0.303	2.548	0.696
p.lay	0.114	0.040	0.116	0.076	0.002	0.971

The mean differences in the life cycle parameters are shown for the three pairs of genotypes: unselected control – highly susceptible, unselected control – refractory, and highly susceptible – refractory. Each mean difference is based on the 9 combinations of group (black, red, green) and environment (uninfected, infected, stressed). A paired t-test was used to test whether the mean difference between the two genotypes was significantly different from zero and the p-value (p) is shown. The abbreviations of the life cycle parameters are defined in Table 4. After correcting for multiple comparisons, 0.05/21 = 0.002, none of differences are statistically significant.

**Figure 2:**
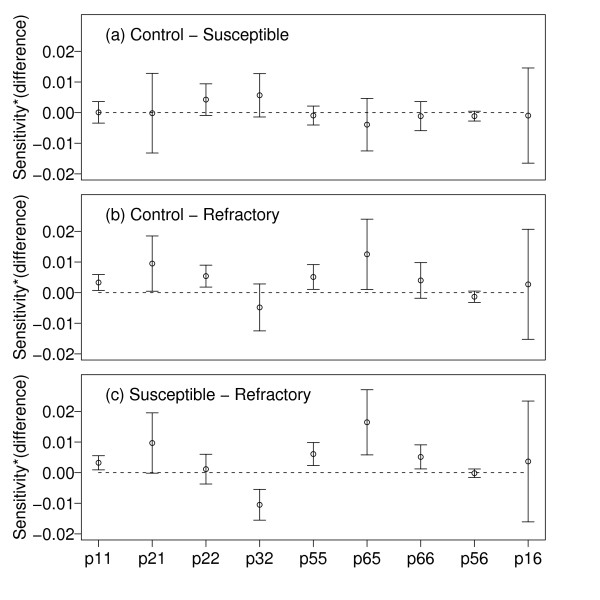
**The life cycle parameters that cause the differences in λ among the 3 genotypes**. The pair wise differences in the matrix entries between the three pairs of genotypes: (a) unselected control – highly susceptible, (b) unselected control – refractory, and (c) highly susceptible – refractory. The subscripts (*i*, *j*) of the matrix entry (p*ij*) refer to the six stages: (1) egg, (2) larvae, (3) pupae, (4) virgin, (5) mated, and (6) gravid females. The differences in the matrix entries are scaled by the sensitivities of the average stage-classified matrix to show how they contribute to differences in λ between each pair of genotypes. For each matrix entry, the mean difference between the two genotypes and the 95% confidence interval are shown for the 9 combinations of group and environment. None of the differences are statistically significant after correcting for multiple comparisons.

From the pair wise differences in Table 3 it is difficult to determine which life cycle parameters are driving the differences in λ because they are in different units. To determine which life cycle parameters contribute to differences in λ between pairs of genotypes we must use the matrix entries instead (see Table 4 and the methods on how the matrix entries are related to the life cycle parameters). In Figure 2, the pair wise differences in the matrix entries are all in the same units of λ after scaling them by the sensitivities (of the average stage-classified matrix). In Figure 2 the emphasis is on the direction and magnitude of the difference in the scaled matrix entry between pairs of genotypes rather than statistical significance (again, none of the 95% confidence intervals in Figure 2 are statistically significant after correcting for multiple comparisons). The lower λ of the refractory genotype was primarily driven by lower egg hatching success (p_21_) and the lower transition of mated to gravid females (p_65_). The transition p_65 _includes both the proportion of females that took a blood meal (p.blood) and the proportion of females that survived blood digestion (p.surv.blood; see Table 4). As we were primarily interested in the latter, we set p.blood equal to one, and recalculated λ and the stage-classified matrices for the 27 combinations. The results were the same indicating that reduced post blood-feeding survival caused the fitness cost of the refractory genotype.

**Table 4:** The ten parameters of the laboratory life cycle of *A. gambiae*.

Abbreviation	Life cycle parameter definition	Contributes to matrix entries (p_ij_)
F	number of eggs produced per female	p_16_
p.lay	proportion of eggs that were laid	p_16_
p.fem	proportion of eggs that were female	p_16_
p.hatch	proportion of eggs that hatched	p_11 _and p_21_
p.pupate	proportion of larvae that reached the pupa stage	p_22 _and p_32_
p.emerge	proportion of pupae that emerged as adults	p_33 _and p_43_
p.mate	proportion of virgin females that were mated	p_44 _and p_54_
p.blood	proportion of mated females that blood fed	p_55 _and p_65_
p.surv.blood	proportion of mated, blood-fed females that survived digesting the blood meal	p_55 _and p_65_
p.surv.ovip	proportion of females that survived oviposition	p_66_, p_56 _and p_16_

The parameter abbreviations were taken from the life cycle in Figure 4. Also shown are the 13 matrix entries (p_ij_) to which each of the 10 life cycle parameters contribute.

### Sensitivity and elasticity analyses of λ

In the previous paragraph, we used the sensitivities to scale the differences in matrix entries between pairs of genotypes to determine which life cycle parameters caused the differences in λ between genotypes. The sensitivity and elasticity analyses also indicate which life cycle parameters have the greatest influence on λ. Such knowledge is valuable for understanding life history evolution in *A. gambiae *and for any strategy seeking to control mosquito population growth.

**Figure 3:**
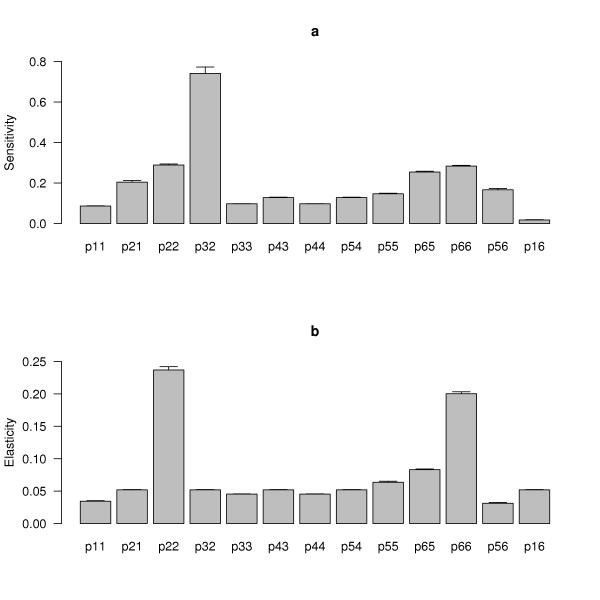
**The contribution of the life cycle parameters to the population growth rate of *A. gambiae***. The sensitivity (a) and the elasticity (b) of λ to the stage-classified matrix entries of *A. gambiae*. For each matrix entry (p*ij*), the subscripts *i *and *j *refer to the six stages: (1) egg, (2) larvae, (3) pupae, (4) virgin females, (5) mated females, and (6) gravid females. For example, p11 is the daily probability that an egg will survive whereas p21 is the daily probability that an egg will hatch and become a larva. For each matrix entry, the mean and standard error are shown for the 27 combinations of group, environment, and genotype.

The sensitivity of λ to the matrix entry p_ij _is ∂λ/∂p_ij_, the derivative of λ with respect to p_ij_. The sensitivities are analogous to the partial regression coefficients of a multiple regression where all the variables are measured in different units (e.g. survival, fertility, probability of blood feeding). For the average stage-classified matrix, the sensitivity of λ to the transition from larva to pupa was the highest (p_32 _= 0.74), followed by sensitivity of λ to larval survival (p_22 _= 0.29), gravid female survival (p_66 _= 0.28), the transition from mated to gravid female (p_65 _= 0.25), and the transition from egg to larva (p_21 _= 0.20; Figure 3).

The elasticity of λ to the matrix entry p_ij _is (p_ij_/λ)*(∂λ/∂p_ij_) and can be interpreted as the proportional contribution of p_ij _to λ. The elasticities are analogous to the partial regression coefficients of a multiple regression where all the variables have been standardized to z-scores (mean 0, standard deviation 1). The elasticity of λ to larval survival was the highest (p_22 _= 0.24), followed by gravid female survival (p_66 _= 0.20; Figure 3). Hence both the sensitivity and the elasticity analysis suggest that larval survival to the pupa stage (p_22 _and p_32_) and gravid female survival (p_66_) have the greatest influence on the lifetime fitness of female *A. gambiae *mosquitoes.

## Discussion

This study shows the utility of using matrix population models to combine multiple life history traits into a single estimate of the population growth rate (λ) when testing for genetic trade-offs between parasite resistance and lifetime fitness. The most important result of this study is that our estimates of λ suggest that a population of refractory mosquitoes will be half the size of a population of highly susceptible mosquitoes in just 23 days. Hence, complete resistance to *Plasmodium *can be costly for *A. gambiae *and may explain why natural populations of mosquitoes maintain genetic variation for malaria resistance. The strategy to combat malaria by replacing natural mosquito populations with transgenic, malaria-resistant mosquitoes will fail if the transgenics carry similar fitness costs.

The population growth rate of the refractory genotype was always lower than that of the highly susceptible genotype (Figure 1). This suggests that the immune mechanisms required for complete refractoriness to *P. yoelii nigeriensis *are costly for *A. gambiae *and would not evolve under the laboratory conditions used in this study. This conclusion is supported by Hurd et al.'s [13] laboratory evolution experiment where mosquitoes fed exclusively on infected mice over 22 generations did not evolve refractoriness. Likewise, two recent population genetic studies estimated the strength of selection on 8 different anti-*Plasmodium *defence genes in the *A. gambiae *species complex and concluded that there was no evidence for strong directional or balancing selection on these genes [26,27]. Such non-significant patterns of selection are expected if the costs of evolving *Plasmodium*-resistance genes are similar to the costs of *Plasmodium *infection. More generally, our results are consistent with numerous studies on other host-parasite systems that have found that the evolution of anti-parasite defence mechanisms in the host comes at the expense of other life history traits [4,6,7].

The conclusions of this study differ from Hurd et al. [13] because we combined all the life cycle parameters into a single measure of fitness, λ. Numerous authors have pointed out the importance of combining the components of fitness (survival, reproduction, development rates) into a measure of lifetime fitness such as λ [11,12,28]. Univariate analyses of multiple fitness components do not provide insight into lifetime fitness because they do not account for the conditional dependence of later expressed components of fitness (e.g. fertility) on those expressed earlier (e.g. survival to reproduce). Furthermore, such analyses are likely to give conflicting results because negative correlations between fitness components are common [10,29]. The ambiguity of this approach is illustrated by the analysis of Hurd et al. [13], which found no clear pattern of differences in fitness components among the three genotypes. Similarly, none of the pair wise t-tests of the 7 life cycle parameters were statistically significant after correcting for multiple comparisons (Table 3). However, after combining the life cycle parameters into a single measure of fitness (λ) we found significant differences among the three genotypes. This study shows that lifetime fitness is the product of many parts and that small, statistically insignificant but consistent differences in these parts can add up to large differences over the course of a life cycle.

Our results are consistent with the only other study to test for genetic trade-offs between malaria resistance and other life history traits in a mosquito [30]. Yan et al. [30] found that their refractory strain of *Aedes aegypti *was smaller, had lower survivorship and laid fewer eggs than the highly susceptible strain in both the presence and absence of the avian malaria parasite, *P. gallinaceum*. In contrast to Yan et al. [30], the major strengths of this study were that we (1) selected the refractory and highly susceptible strains from the same population, (2) included unselected control genotypes allowing us to rule out inbreeding effects, and (3) repeated the experiment three times (i.e. the black, red and green groups). One limitation of this study is that there was no replication of mice within the 9 combinations of group and environment. It is therefore possible that the significant group:environment interaction on λ (model 5 in Table 1) was caused by random variation among mice. It is well known, for example, that the gametocyte density in the vertebrate host influences the infectivity of the blood meal and the subsequent oocyst load in the mosquito [31]. Fortunately, because the three genotypes were blocked by the factor 'mouse', the limitations of the experimental design do not affect the conclusion that the population growth rate of the refractory genotype is lower than that of the other two genotypes.

The life cycle parameters that reduced λ the most for the refractory genotype were post blood-feeding survival and hatching success (Figure 2). The lower post blood-feeding survival suggests that the refractory mosquitoes evolved immune responses that harm both "self" and "non-self" (i.e. autoimmunity costs; [2]). For example, following an infected blood meal, *Anopheles *females upregulate expression of nitric oxide synthase producing levels of nitric oxide [32] that limit ookinete development [33] but may also be toxic for the mosquito [34]. Similarly, the phenoloxidase cascade responsible for the melanization of oocysts in the midgut produces phenol by-products that may be cytotoxic for the mosquito [35,36]. The induction of the melanization response in gravid *A. gambiae *females also reduces the deposition of protein (e.g. vitellin) in the eggs [37]. Studies with *Plasmodium*-infected *Anopheles *females have shown reduced vitellin provisioning of eggs, which may result in lower hatch rates [38,39]. Hence, a trade-off between vitellin egg provisioning and an upregulated immune system post-blood feeding in refractory females is one explanation for their lower egg hatch rates.

We found no evidence that *Plasmodium *infection reduced the population growth rate of *A. gambiae*. The main effect of environment (presence versus absence of *Plasmodium*) was not statistically significant and explained only 8.2% of the variation in λ (Table 2). In contrast, the main effect of genotype accounted for 21.7% of the variation in λ (Table 2). Although it has been repeatedly shown that *Plasmodium *reduces egg production in *Anopheles *mosquitoes [15-17] including the Keele population from which the refractory and highly susceptible genotypes were selected by Hurd et al. [13], our sensitivity and elasticity analyses (Figure 3) show that λ is minimally affected by changes in fertility. The effect of *Plasmodium *on mosquito survival is more controversial [18]. Environmental factors can also influence the virulence of the mosquito-*Plasmodium *interaction. For example, Lambrechts et al. [40] showed that *A. stephensi *infected with *P. yoelii yoelii *suffer more than uninfected individuals when fed on low glucose levels. Other studies have shown that *Plasmodium*-induced mortality is influenced by humidity, temperature, diet, larval density, and bacterial infection (reviewed in [18]). How this environmental variation structures the virulence of the mosquito-*Plasmodium *interaction in the field is an open question. In this study, the stressed environment was supposed to mimic field conditions and included cold temperatures, reduced sugar, and cage shaking to induce flight. However, because the experiment did not include an uninfected and stressed treatment it was not possible to determine whether environmental stress increased or decreased the cost of infection.

## Conclusion

In this laboratory population of *A. gambiae*, the population growth rate of the malaria-resistant mosquito genotype was significantly lower than that of the highly susceptible and unselected control genotypes regardless of whether the mosquitoes were fed on *Plasmodium*-infected or uninfected blood. This cost of refractoriness was driven by lower post-blood feeding survival and egg hatching of refractory females. If the costs of *Plasmodium *refractoriness in the field are higher than the costs of *Plasmodium *infection, this may explain why natural populations of *A. gambiae *are not uniformly resistant to malaria parasites [23]. With respect to the transgenic strategy for eradicating malaria, this study has several important implications. Our sensitivity and elasticity analyses show that the insertion of any anti-*Plasmodium *defence genes into transgenic mosquitoes must avoid reducing larval survival to the pupa stage at all costs as this life history trait has the greatest influence on λ. Anti-*Plasmodium *genes (e.g. nitric oxide synthase, or enzymes in the phenoloxidase cascade) that increase the production of toxins (e.g. nitric oxide, phenol by-products) and incur autoimmunity costs may do more harm than good [2]. Fortunately, there is at least one example of a transgenic strain of *A. stephensi*, which expresses the SM1 peptide in the midgut, that is resistant to the rodent malaria parasite *P. berghei*, that has a selective advantage over non-transgenic mosquitoes when fed on *P. berghei*-infected mice, and that does not appear to have a selective disadvantage when fed on uninfected mice [41]. If similar transgenic mosquito strains can be created for human *Plasmodium *parasites there is room for optimism that the transgenic strategy may yet succeed. However, if transgenic mosquitoes carry similar costs of being refractory to those measured in the present study they would be unable to replace natural populations [14,42,43].

## Methods

### Experimental design of the study by Hurd et al. (2005)

Hurd et al. [13] created three replicate selection experiments, referred to as the black, red and green groups. All three groups were sampled from the outbred Keele population, which was created by the balanced interbreeding of four laboratory strains of *A. gambiae *sensu stricto: the KIL, G3, Zan U and Ifakara strains (see [13]). For each group, there were three selection lines: (1) selection for zero malaria oocysts in the mid gut of female mosquitoes 7 days after an infected blood meal to create the refractory line, (2) selection for high numbers of oocysts in the mosquito mid gut after an infected blood meal to create the susceptible line, and (3) random selection after an uninfected blood meal to create an unselected control line. After 10 generations of selection, the mean oocyst load of the refractory lines (12.1 oocysts per mosquito) was much lower than that of the highly susceptible and control lines (99.2 and 84.4 oocysts per mosquito, respectively). Hurd et al. [13] compared the fitness of the unselected control lines with the outbred Keele population to confirm that no inbreeding depression had occurred.

For each of the 3 groups (black, red, green), Hurd et al. [13] fed the 3 genotypes (unselected control, refractory, highly susceptible) on one uninfected mouse and one *P. y. nigeriensis*-infected mouse. The uninfected and malaria-infected mice represent the two different blood-feeding environments where the *Plasmodium *parasite was either absent or present. For each of the 3 groups, a third environment was created where the 3 genotypes were stressed (see [13]) after feeding on one infected mouse. A different mouse was used for each of the 9 combinations of group and environment so that a total of 9 mice were used in the experiment (3 uninfected and 6 infected). For each of the 9 combinations of group and environment, the 3 genotypes were fed on the same mouse.

For each of the 27 combinations of group, environment, and genotype, Hurd et al. [13] measured a number of life history traits starting with ~100 blood fed *A. gambiae *female mosquitoes including: (1) the proportion of female mosquitoes that took a blood meal, (2) the proportion of females that died while digesting the blood meal (3) the proportion of female mosquitoes that died during oviposition, (4) the number of eggs produced per female, (5) the proportion of eggs that were laid, (6) the proportion of eggs that hatched, and (7) the proportion of larvae that reached the pupae stage.

### Stage-classified life cycle of *A. gambiae*

**Figure 4:**
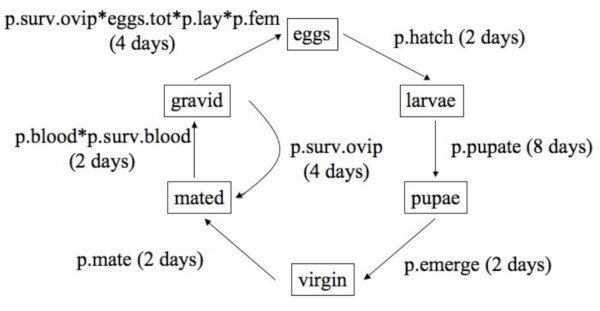
**The laboratory life cycle of *Anopheles gambiae***. There are six stages in the life cycle of *A. gambiae*: eggs, larvae, pupae, virgins, mated, and gravid females. All stages refer to females only. At the end of the 4 day oviposition period, the gravid females return to the mated state. The parameters of the life cycle include: the proportion of eggs that hatch (p.hatch), the proportion of larvae that pupate (p.pupate), the proportion of pupae that emerge as virgins (p.emerge), the proportion of virgins that are mated (p.mate), the proportion of mated females that take a blood meal (p.blood) and that survive digesting the blood meal (p.surv.blood), the proportion of females that survive the oviposition period (p.surv.ovip), the total number of eggs produced (eggs.tot), the proportion of eggs that are laid (p.lay), and the proportion of female eggs (p.fem).

We classified the life cycle of *A. gambiae *into six stages: (1) eggs, (2) larvae, (3) pupae, (4) virgin, (5) mated, and (6) gravid females (Figure 4). All stages refer to females only. Under laboratory conditions, the durations of these 6 stages were 2, 8, 2, 2, 2, and 4 days, respectively (Figure 4). In the field, *A. gambiae *can lay up to 12 batches of eggs [44]. *A. gambiae *females produce a batch of eggs every 3 days when given regular access to blood meals [45]. For the purpose of this model we assumed that once a gravid female has laid her eggs, she is similar in state to a mated female (i.e. she has sperm and is motivated to search for a blood meal). The arrow from the gravid to the mated state in Figure 4 reflects that females can lay multiple batches of eggs.

There are 10 parameters in Figure 4 (defined in Table 4) that describe the transitions between the six stages of the life cycle. We used the data from Hurd et al. [13] to estimate 7 of these life cycle parameters for each of the 27 combinations of group, environment, and genotype (see Additional files 1 and 2). For each of the 27 combinations, Hurd et al. [13] obtained one estimate of fertility from the first batch of eggs and we used this estimate for all batches. Hence our model assumes that female fertility was constant over time. Hurd et al. [13] did not estimate the other three life cycle parameters: the probability of successful pupation (p.emerge), the probability of mating (p.mate), and the sex ratio of the offspring (p.fem), so, for every experimental combination, we set these parameters to 0.9, 0.9 and 0.5, respectively. The justification for these parameter values was as follows. In the selection lines used in this experiment, the percentage of pupae that emerge as adults is ~90% (Maarten Voordouw, personal observation), hence we set p.emerge to 0.9. Hurd et al. [13] found that female insemination was greater than 90% after 3 days of mating, hence we set p.mate to 0.9. A sex ratio of 0.5 is a reasonable estimate for *A. gambiae*, which has sex chromosomes; hence we set p.fem to 0.5. Because these life cycle parameters were kept constant they cannot contribute to differences in λ among the factors of interest (group, environment, and genotype).

### The characteristic equation to estimate λ of *A. gambiae*

**Figure 5:**
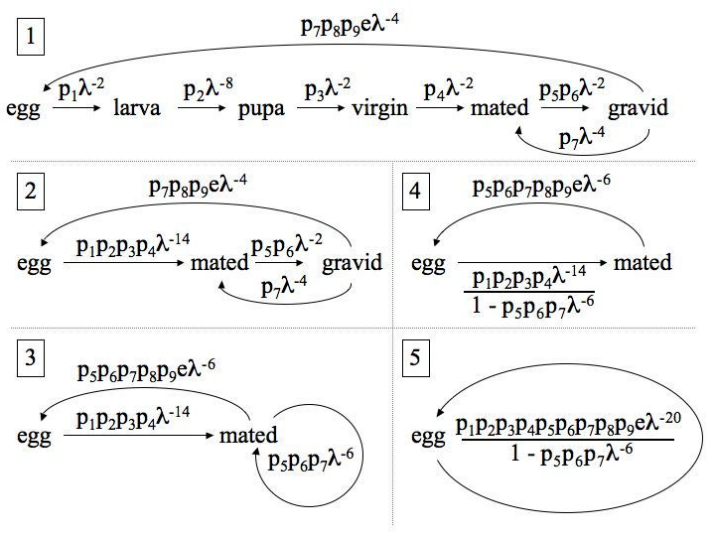
**The characteristic equation of the life cycle of *A. gambiae***. The life cycle graph is reduced in five consecutive steps. In panel 5, the equation is set to 1 and λ^20 ^- p_5 _*p_6 _*p_7 _* λ^14 ^- p_1 _*p_2 _*p_3 _*p_4 _*p_5 _*p_6 _*p_7 _*p_8 _*p_9 _*e = 0 is the characteristic equation that can be solved for λ. The ten life cycle parameters are p.hatch (p_1_), p.pupate (p_2_), p.emerge (p_3_), p.mate (p_4_), p.blood (p_5_), p.surv.blood (p_6_), p.surv.ovip (p_7_), p.lay (p_8_), p.fem (p_9_), and eggs.tot (e).



To model stage-classified population growth over discrete time, we chose a time interval of 1 day. Hence, all estimates of the population growth rate (λ) will have units of day^-1^. The life cycle graph in Figure 4 represents a system of linear difference equations that describe the changes in abundance of the six *A. gambiae *stages over time. These linear difference equations can be written as a stage-classified matrix where λ is the dominant eigenvalue. Alternatively, these equations can be transformed into a power series in λ using the *z*-transform [12]. This power series is known as the characteristic equation of the life cycle and it can be solved numerically for λ [12]. To obtain the characteristic equation, we created the *z*-transformed life cycle graph (panel 1 in Figure 5), where the transitions between stage *i *and stage *i*+1 are time-lagged by dividing them by λ^Ti ^and T_i _is the duration of stage *i*. We reduced the *z*-transformed life cycle graph to its simplest form (panel 5 in Figure 5) following the permissible reductions in Caswell [12] to obtain the characteristic equation:

We solved the characteristic equation for λ for each of the 27 combinations of group, environment, and genotype (see Additional file 2).

### Stage-classified matrix of *A. gambiae*

We created a stage-classified matrix for the six stages of *A. gambiae *with a projection interval of one day. For each stage (hereafter referred to as stage *i*), we calculated the daily probability of survival, σ_i _= (p_i_)^(1/T_i_), where p_i _is the proportion of stage *i *individuals that reach the next stage (i.e. the life cycle parameters in Figure 4 and Table 4), and T_i _is the duration of stage *i *in days. We assumed that each individual spends exactly T_i _days in stage *i*, that all individuals in the last day of that stage (T_i _- 1) graduate to the next stage, and that the age distribution within the stage is stable (equation 6.100 in [12]). These assumptions and our estimates of λ (from the characteristic equation) allowed us to calculate γ_i_, the proportion of individuals in the last day of stage *i *that graduate to the next stage (equation 6.101 in [12]; see Additional file 3). We used our estimates of σ_i _and γ_i _to calculate G_i _and P_i_, which are the daily probabilities that an individual either graduates to the next stage or remains in the current stage (using equations 6.97 and 6.98 in [12]; see Additional file 3). The values of p_i_, T_i_, σ_i_, γ_i_, G_i _and P_i _are shown in Additional file 3. We created a stage-classified matrix for each of the 27 combinations of group, environment, and genotype (see Additional file 4). We used these matrices to conduct sensitivity and elasticity analyses following Caswell [12].

### Statistical methods

For each of the 9 combinations of group (black, red, green) and environment (uninfected, infected, infected & stressed), Hurd et al. [13] blocked the three genotypes (unselected control, refractory, highly susceptible) by feeding them on the same mouse (see Additional file 1). This was done to control for variation among mice in gametocyte density and hematocrit levels.

All statistical analyses were done in R version 2.7.0. The λ values were normally distributed. We modelled λ as a linear function of the three factors: group, environment, genotype and their interactions. We ran all possible models except for the full factorial model because there were not enough degrees of freedom. We used Akaike's information criterion (AIC) to guide model selection. The best model was within 1 unit of the lowest AIC score and had the fewest number of parameters. For the best model, we used F-tests to test the significance of the factors and interactions included in the model. For the factor genotype, we used two planned contrasts to test two different hypotheses about λ. The first contrast compares the mean λ of the unselected control genotype with that of the selected genotypes (i.e., the refractory and highly susceptible genotypes combined) to test the hypothesis that selection reduced λ (e.g., due to inbreeding). The second contrast compares the mean λ of the refractory genotype with that of the highly select genotype to test the hypothesis that the evolution of *Plasmodium*-resistance reduced λ (e.g. due to pleiotropy).

To determine which life cycle parameters were causing the differences in λ among genotypes we used pair wise t-tests to compare the 7 life cycle parameters for each pair of genotypes (unselected control – refractory, unselected control – highly susceptible, highly susceptible – refractory). We did not compare the other 3 life cycle parameters (p.emerge, p.mate, and p.fem in Table 4) because these were constant among genotypes. To correct for multiple comparisons we set the significance level at 0.05/21 = 0.002. The utility of this approach to determine which life cycle parameters are causing the differences in λ is limited because the parameters are in different units. To account for this problem, we also compared 9 of the 13 matrix entries after scaling them by the sensitivities of the average stage-classified matrix. This scaling ensures that all the matrix entries are in units of λ. For the pair wise comparison of the scaled matrix entries, the emphasis is on the direction and magnitude of the difference between pairs of genotypes rather than the statistical significance. We did not compare the matrix entries p_33_, p_43_, p_44_, and p_54 _because these are derived from the life cycle parameters p.emerge and p.mate (see Table 4), which are constants and therefore cannot contribute to differences in λ among genotypes.

## Authors' contributions

MJV conceived the idea to re-analyze the data of Hurd et al. (2005) using matrix population models, analyzed and interpreted the data, and wrote the manuscript. PT conducted the original experiment and collected the data published in Hurd et al. (2005). BA and HH helped to interpret the data and write the manuscript. All authors read and approved the final manuscript.

## Supplementary Material

Additional file 1 ** Additional Table 1**. The life table data of *Anopheles gambiae *from the study by Hurd et al. (2005). Click here for file

Additional file 2 ** Additional Table 2**. The laboratory life cycle parameters of *A. gambiae *(see Figure 4). Click here for file

Additional file 3 ** Additional Table 3**. The matrix entries for the stage-classified population matrices of *A. gambiae*. Click here for file

Additional file 4 ** Additional Table 4**. The stage-classified population matrices of *A. gambiae*. Click here for file
