# Supplementary material for: Rodent malaria-resistant strains of the mosquito, Anopheles gambiae, have slower population growth than -susceptible strains
Source: BMC Evol Biol. 2009 Apr 20;9:76. doi: 10.1186/1471-2148-9-76 (PMC2675531; doi:10.1186/1471-2148-9-76)
Supplement: Additional file 1 — Additional Table 1. The life table data of Anopheles gambiae from the study by Hurd et al. (2005). [file 1471-2148-9-76-S1.doc]

Additional Table 1. The life table data of *Anopheles gambiae* from the study by Hurd et al. (2005). Shown are the 3 replicate selection experiments (black, red, and green groups), the 3 environments (mosquitoes were fed on uninfected mice, *Plasmodium yoelii nigeriensis*-infected mice, and *P. y. nigeriensis*-infected mice and were subsequently stressed), and the 3 genotypes (unselected control, *P. y. nigeriensis*-refractory, and *P. y. nigeriensis*-susceptible). Each of the 9 combinations of group and environment used a different mouse (A, B, C, D, E, F, G, H, I). The three genotypes were fed on the same mouse. For each of the 27 combinations of group, environment, and genotype, the total number of females in the blood feeding cage (c.total), the number of females that took a blood meal (c.feed), the number of blood fed females transferred to tubes to measure haematin excretion (t.total), and the number of females that were alive (t.alive) and dead (t.dead) after two days in the tubes are shown. All females that were alive after two days in the tubes (t.alive) were transferred to individual oviposition cups to measure egg production. Also shown are the number of females that were alive (o.alive) and dead (o.dead) after 4 days in the oviposition cups, the total number of eggs produced (e.tot = laid and retained eggs), the total number of eggs laid (e.laid), the total number of eggs that hatched (n.hatch), the total number of larvae that were selected to be reared (n.rear), and the total number of pupae produced (n.pupae). Per capita reproduction was calculated by dividing the reproductive columns (e.tot, e.laid, n.hatch, n.rear, n.pupae) by the number of females that were allowed to oviposit (t.alive).

| group | environment | genotype | mouse | c.total | c.feed | t.total | t.alive | t.dead | o.alive | o.dead | e.tot | e.laid | n.hatch | n.rear | n.pupae |
| --- | --- | --- | --- | --- | --- | --- | --- | --- | --- | --- | --- | --- | --- | --- | --- |
| black | uninfected | control | A | 80 | 60 | 60 | 49 | 11 | 48 | 1 | 2473 | 1367 | 833 | 657 | 492 |
| black | uninfected | refractory | A | 80 | 56 | 56 | 53 | 3 | 49 | 4 | 3803 | 1961 | 830 | 643 | 486 |
| black | uninfected | susceptible | A | 80 | 67 | 67 | 59 | 8 | 55 | 4 | 3542 | 1991 | 980 | 789 | 574 |
| black | infected | control | B | 116 | 114 | 80 | 73 | 7 | 67 | 6 | 5764 | 4730 | 2477 | 1749 | 1616 |
| black | infected | refractory | B | 112 | 93 | 80 | 74 | 6 | 67 | 7 | 7285 | 4157 | 1914 | 1211 | 918 |
| black | infected | susceptible | B | 120 | 116 | 80 | 76 | 4 | 72 | 4 | 5734 | 4404 | 2887 | 1638 | 729 |
| black | stressed | control | C | 111 | 103 | 80 | 60 | 20 | 58 | 2 | 3968 | 3457 | 2512 | 1515 | 1330 |
| black | stressed | refractory | C | 107 | 82 | 80 | 54 | 26 | 37 | 17 | 2301 | 1881 | 688 | 600 | 425 |
| black | stressed | susceptible | C | 120 | 107 | 80 | 60 | 20 | 56 | 4 | 4585 | 3893 | 1314 | 919 | 720 |
| red | uninfected | control | D | 100 | 100 | 80 | 80 | 0 | 76 | 4 | 5450 | 3610 | 1946 | 1348 | 1291 |
| red | uninfected | refractory | D | 95 | 70 | 70 | 53 | 17 | 48 | 5 | 3083 | 2138 | 849 | 695 | 648 |
| red | uninfected | susceptible | D | 105 | 93 | 80 | 77 | 3 | 74 | 3 | 6487 | 4028 | 2125 | 1415 | 1296 |
| red | infected | control | E | 96 | 94 | 80 | 59 | 21 | 55 | 4 | 1932 | 1267 | 514 | 472 | 433 |
| red | infected | refractory | E | 80 | 72 | 72 | 56 | 16 | 53 | 3 | 3288 | 2027 | 959 | 679 | 590 |
| red | infected | susceptible | E | 84 | 81 | 80 | 58 | 22 | 52 | 6 | 3059 | 2160 | 1583 | 847 | 585 |
| red | stressed | control | F | 80 | 45 | 45 | 45 | 0 | 30 | 15 | 1160 | 907 | 553 | 446 | 290 |
| red | stressed | refractory | F | 80 | 58 | 58 | 41 | 17 | 35 | 6 | 1417 | 769 | 367 | 281 | 163 |
| red | stressed | susceptible | F | 80 | 68 | 68 | 68 | 0 | 64 | 4 | 3304 | 2240 | 1342 | 909 | 559 |
| green | uninfected | control | G | 108 | 92 | 80 | 70 | 10 | 64 | 6 | 4949 | 3271 | 1369 | 784 | 693 |
| green | uninfected | refractory | G | 111 | 99 | 80 | 67 | 13 | 64 | 3 | 5169 | 2681 | 669 | 547 | 363 |
| green | uninfected | susceptible | G | 120 | 96 | 80 | 72 | 8 | 70 | 2 | 5979 | 1901 | 887 | 594 | 435 |
| green | infected | control | H | 112 | 87 | 80 | 68 | 12 | 53 | 15 | 3919 | 2651 | 1556 | 965 | 830 |
| green | infected | refractory | H | 117 | 82 | 80 | 56 | 24 | 39 | 17 | 2845 | 679 | 455 | 337 | 302 |
| green | infected | susceptible | H | 106 | 86 | 80 | 69 | 11 | 56 | 13 | 2720 | 1077 | 551 | 363 | 247 |
| green | stressed | control | I | 113 | 98 | 80 | 60 | 20 | 52 | 8 | 2304 | 1599 | 989 | 690 | 565 |
| green | stressed | refractory | I | 115 | 105 | 80 | 33 | 47 | 30 | 3 | 1679 | 1377 | 653 | 494 | 376 |
| green | stressed | susceptible | I | 94 | 86 | 80 | 62 | 18 | 57 | 5 | 2857 | 1304 | 964 | 616 | 501 |
