# Supplementary material for: Rodent malaria-resistant strains of the mosquito, Anopheles gambiae, have slower population growth than -susceptible strains
Source: BMC Evol Biol. 2009 Apr 20;9:76. doi: 10.1186/1471-2148-9-76 (PMC2675531; doi:10.1186/1471-2148-9-76)
Supplement: Additional file 2 — Additional Table 2. The laboratory life cycle parameters of A. gambiae (see Figure 4). [file 1471-2148-9-76-S2.doc]

Additional Table 2. The laboratory life cycle parameters of *A. gambiae* (see Figure 4). The life cycle parameters were estimated for each of the 27 combinations of group, environment and genotype using the life table data in Additional file 1. Shown are the proportion of eggs that hatched (p.hatch), the proportion of larvae that reached the pupa stage (p.pupate), the proportion of mated females that blood fed (p.blood), the proportion of females that survived the blood meal (p.surv.blood), the proportion of females that survived oviposition (p.surv.ovip), the number of eggs produced per ovipositing female (eggs.tot), the proportion of eggs that were laid (p.lay), and the geometric rate of increase (λ). The other three life cycle parameters are not shown because they are constant for the 27 experimental combinations: proportion of females that emerged (p.emerge = 0.9), proportion of females that mated (p.mate = 0.9), and proportion of eggs that are female (p.fem = 0.5).

| group | environment | genotype | mouse | p.hatch | p.pupate | p.blood | p.surv.blood | p.surv.ovip | eggs.tot | p.lay | λ |
| --- | --- | --- | --- | --- | --- | --- | --- | --- | --- | --- | --- |
| black | uninfected | control | A | 0.609 | 0.749 | 0.750 | 0.817 | 0.980 | 50.5 | 0.553 | 1.083 |
| black | uninfected | refractory | A | 0.423 | 0.756 | 0.700 | 0.946 | 0.925 | 71.8 | 0.516 | 1.081 |
| black | uninfected | susceptible | A | 0.492 | 0.728 | 0.838 | 0.881 | 0.932 | 60.0 | 0.562 | 1.091 |
| black | infected | control | B | 0.524 | 0.924 | 0.983 | 0.913 | 0.918 | 79.0 | 0.821 | 1.150 |
| black | infected | refractory | B | 0.460 | 0.758 | 0.830 | 0.925 | 0.905 | 98.4 | 0.571 | 1.114 |
| black | infected | susceptible | B | 0.656 | 0.445 | 0.967 | 0.950 | 0.947 | 75.4 | 0.768 | 1.125 |
| black | stressed | control | C | 0.727 | 0.878 | 0.928 | 0.750 | 0.967 | 66.1 | 0.871 | 1.142 |
| black | stressed | refractory | C | 0.366 | 0.708 | 0.766 | 0.675 | 0.685 | 42.6 | 0.817 | 1.031 |
| black | stressed | susceptible | C | 0.338 | 0.783 | 0.892 | 0.750 | 0.933 | 76.4 | 0.849 | 1.100 |
| red | uninfected | control | D | 0.539 | 0.958 | 1.000 | 1.000 | 0.950 | 68.1 | 0.662 | 1.147 |
| red | uninfected | refractory | D | 0.397 | 0.932 | 0.737 | 0.757 | 0.906 | 58.2 | 0.693 | 1.078 |
| red | uninfected | susceptible | D | 0.528 | 0.916 | 0.886 | 0.963 | 0.961 | 84.2 | 0.621 | 1.139 |
| red | infected | control | E | 0.406 | 0.917 | 0.979 | 0.738 | 0.932 | 32.7 | 0.656 | 1.071 |
| red | infected | refractory | E | 0.473 | 0.869 | 0.900 | 0.778 | 0.946 | 58.7 | 0.616 | 1.098 |
| red | infected | susceptible | E | 0.733 | 0.691 | 0.964 | 0.725 | 0.897 | 52.7 | 0.706 | 1.105 |
| red | stressed | control | F | 0.610 | 0.650 | 0.563 | 1.000 | 0.667 | 25.8 | 0.782 | 1.029 |
| red | stressed | refractory | F | 0.477 | 0.580 | 0.725 | 0.707 | 0.854 | 34.6 | 0.543 | 1.021 |
| red | stressed | susceptible | F | 0.599 | 0.615 | 0.850 | 1.000 | 0.941 | 48.6 | 0.678 | 1.103 |
| green | uninfected | control | G | 0.419 | 0.884 | 0.852 | 0.875 | 0.914 | 70.7 | 0.661 | 1.107 |
| green | uninfected | refractory | G | 0.250 | 0.664 | 0.892 | 0.838 | 0.955 | 77.1 | 0.519 | 1.068 |
| green | uninfected | susceptible | G | 0.467 | 0.732 | 0.800 | 0.900 | 0.972 | 83.0 | 0.318 | 1.079 |
| green | infected | control | H | 0.587 | 0.860 | 0.777 | 0.850 | 0.779 | 57.6 | 0.676 | 1.093 |
| green | infected | refractory | H | 0.670 | 0.896 | 0.701 | 0.700 | 0.696 | 50.8 | 0.239 | 1.019 |
| green | infected | susceptible | H | 0.512 | 0.680 | 0.811 | 0.863 | 0.812 | 39.4 | 0.396 | 1.041 |
| green | stressed | control | I | 0.619 | 0.819 | 0.867 | 0.750 | 0.867 | 38.4 | 0.694 | 1.081 |
| green | stressed | refractory | I | 0.474 | 0.761 | 0.913 | 0.413 | 0.909 | 50.9 | 0.820 | 1.053 |
| green | stressed | susceptible | I | 0.739 | 0.813 | 0.915 | 0.775 | 0.919 | 46.1 | 0.456 | 1.089 |
