# Supplementary material for: Rodent malaria-resistant strains of the mosquito, Anopheles gambiae, have slower population growth than -susceptible strains
Source: BMC Evol Biol. 2009 Apr 20;9:76. doi: 10.1186/1471-2148-9-76 (PMC2675531; doi:10.1186/1471-2148-9-76)
Supplement: Additional file 3 — Additional Table 3. The matrix entries for the stage-classified population matrices of A. gambiae. [file 1471-2148-9-76-S3.doc]

**Additional** **Table 3. The matrix entries for the stage-classified population matrices of *A. gambiae*.** The matrix entries were estimated for each of the 27 experimental combinations of group, environment, and genotype using the life cycle parameters in Additional file 2. Let stage *i* denote any of the six stages in the life cycle of *A. gambiae*: eggs, larvae, pupae, virgin, mated, and gravid females. The proportion of individuals in stage *i* that reached the next stage (p_i_; equivalent to the life cycle parameters in Additional file 2), the duration of the stage in days (T_i_), and the population growth rate (λ) were used to calculate the daily probability of survival for that stage (σ_i_), the proportion of individuals in the last day of that stage (γ_i_), and the daily probability that an individual either remains in that stage (P_i_) or graduates to the next stage (G_i_). For stage *i*, σ_i_ = (p_i_)^(1/Ti)^, γ_i_ = (σ_i_/λ^Ti^ – σ_i_/λ^(Ti – 1)^)/(σ_i_/λ^Ti^ – 1), P_i_ = σ_i_*(1 – γ_i_), and G_i_ = σ_i_*γ_i_, respectively (equations 6.101, 6.97, and 6.98 in Caswell 2001). For example, for the black, uninfected, control genotype, the proportion of eggs that reached the larval stage p_eggs_ = 0.609, T_eggs_ = 2 days, and λ = 1.083 day^-1^. The daily survival rate σ_eggs_ = (p_eggs_)^(1/Teggs)^ = 0.609^(1/2 days)^ = 0.781 day^-1^, the proportion of eggs in the last day of the egg stage γ_eggs_ = (σ_eggs_/λ^Teggs^ – σ_eggs_/λ^(Teggs – 1)^)/(σ_eggs_/λ^Teggs^ – 1) = (0.781/1.083^2^ – 0.781/1.083^(2 – 1)^)/(0.781/1.083^2^ – 1) = 0.419, the daily probability that an egg remains in the egg stage P_eggs_ = σ_eggs_*(1 – γ_eggs_) = 0.781*(1 – 0.419) = 0.454 day^-1^, and the daily probability that an egg reaches the larval stage G_eggs_ = σ_eggs_*γ_eggs_ = 0.781*0.419 = 0.327 day^-1^. The fertility of gravid females is calculated as F_gravid_ = G_gravid_*eggs.tot*p.lay*p.fem, where G_gravid_ is the probability that a gravid female survives oviposition and reaches the mated stage, eggs.tot is the total number of eggs produced per female, p.lay is the proportion of eggs laid, and p.fem is the proportion of female eggs.

| stage | group | environment | genotype | λ  (day^-1^) | T_eggs_  (days) | p_eggs_  (p.hatch) | σ_eggs_  (day^-1^) | γ_eggs_ | P_eggs_  (day^-1^) | G_eggs_  (day^-1^) |
| --- | --- | --- | --- | --- | --- | --- | --- | --- | --- | --- |
| eggs | black | uninfected | control | 1.083 | 2 | 0.609 | 0.781 | 0.419 | 0.454 | 0.327 |
| eggs | black | uninfected | refractory | 1.081 | 2 | 0.423 | 0.651 | 0.376 | 0.406 | 0.244 |
| eggs | black | uninfected | susceptible | 1.091 | 2 | 0.492 | 0.702 | 0.391 | 0.427 | 0.275 |
| eggs | black | infected | control | 1.150 | 2 | 0.524 | 0.724 | 0.386 | 0.444 | 0.280 |
| eggs | black | infected | refractory | 1.114 | 2 | 0.460 | 0.679 | 0.379 | 0.422 | 0.257 |
| eggs | black | infected | susceptible | 1.125 | 2 | 0.656 | 0.810 | 0.419 | 0.471 | 0.339 |
| eggs | black | stressed | control | 1.142 | 2 | 0.727 | 0.852 | 0.427 | 0.488 | 0.364 |
| eggs | black | stressed | refractory | 1.031 | 2 | 0.366 | 0.605 | 0.370 | 0.381 | 0.224 |
| eggs | black | stressed | susceptible | 1.100 | 2 | 0.338 | 0.581 | 0.346 | 0.380 | 0.201 |
| eggs | red | uninfected | control | 1.147 | 2 | 0.539 | 0.734 | 0.390 | 0.448 | 0.287 |
| eggs | red | uninfected | refractory | 1.078 | 2 | 0.397 | 0.630 | 0.369 | 0.398 | 0.232 |
| eggs | red | uninfected | susceptible | 1.139 | 2 | 0.528 | 0.726 | 0.389 | 0.443 | 0.283 |
| eggs | red | infected | control | 1.071 | 2 | 0.406 | 0.637 | 0.373 | 0.399 | 0.238 |
| eggs | red | infected | refractory | 1.098 | 2 | 0.473 | 0.688 | 0.385 | 0.423 | 0.265 |
| eggs | red | infected | susceptible | 1.105 | 2 | 0.733 | 0.856 | 0.437 | 0.482 | 0.374 |
| eggs | red | stressed | control | 1.029 | 2 | 0.610 | 0.781 | 0.431 | 0.444 | 0.337 |
| eggs | red | stressed | refractory | 1.021 | 2 | 0.477 | 0.691 | 0.404 | 0.412 | 0.279 |
| eggs | red | stressed | susceptible | 1.103 | 2 | 0.599 | 0.774 | 0.412 | 0.455 | 0.319 |
| eggs | green | uninfected | control | 1.107 | 2 | 0.419 | 0.647 | 0.369 | 0.408 | 0.239 |
| eggs | green | uninfected | refractory | 1.068 | 2 | 0.250 | 0.500 | 0.319 | 0.340 | 0.159 |
| eggs | green | uninfected | susceptible | 1.079 | 2 | 0.467 | 0.683 | 0.388 | 0.418 | 0.265 |
| eggs | green | infected | control | 1.093 | 2 | 0.587 | 0.766 | 0.412 | 0.450 | 0.316 |
| eggs | green | infected | refractory | 1.019 | 2 | 0.670 | 0.819 | 0.446 | 0.454 | 0.365 |
| eggs | green | infected | susceptible | 1.041 | 2 | 0.512 | 0.715 | 0.407 | 0.424 | 0.291 |
| eggs | green | stressed | control | 1.081 | 2 | 0.619 | 0.786 | 0.421 | 0.455 | 0.331 |
| eggs | green | stressed | refractory | 1.053 | 2 | 0.474 | 0.689 | 0.395 | 0.416 | 0.272 |
| eggs | green | stressed | susceptible | 1.089 | 2 | 0.739 | 0.860 | 0.441 | 0.480 | 0.379 |
|  |  |  |  |  |  |  |  |  |  |  |
| stage | group | environment | genotype | λ  (day^-1^) | T_larvae_  (days) | p_larvae_  (p.pupate) | σ_larvae_  (day^-1^) | γ_larvae_ | P_larvae_  (day^-1^) | G_larvae_  (day^-1^) |
| larvae | black | uninfected | control | 1.083 | 8 | 0.749 | 0.964 | 0.080 | 0.887 | 0.078 |
| larvae | black | uninfected | refractory | 1.081 | 8 | 0.756 | 0.966 | 0.081 | 0.887 | 0.079 |
| larvae | black | uninfected | susceptible | 1.091 | 8 | 0.728 | 0.961 | 0.077 | 0.887 | 0.074 |
| larvae | black | infected | control | 1.150 | 8 | 0.924 | 0.990 | 0.070 | 0.921 | 0.069 |
| larvae | black | infected | refractory | 1.114 | 8 | 0.758 | 0.966 | 0.072 | 0.896 | 0.070 |
| larvae | black | infected | susceptible | 1.125 | 8 | 0.445 | 0.904 | 0.051 | 0.857 | 0.047 |
| larvae | black | stressed | control | 1.142 | 8 | 0.878 | 0.984 | 0.070 | 0.915 | 0.069 |
| larvae | black | stressed | refractory | 1.031 | 8 | 0.708 | 0.958 | 0.095 | 0.867 | 0.091 |
| larvae | black | stressed | susceptible | 1.100 | 8 | 0.783 | 0.970 | 0.077 | 0.895 | 0.075 |
| larvae | red | uninfected | control | 1.147 | 8 | 0.958 | 0.995 | 0.072 | 0.923 | 0.072 |
| larvae | red | uninfected | refractory | 1.078 | 8 | 0.932 | 0.991 | 0.091 | 0.901 | 0.091 |
| larvae | red | uninfected | susceptible | 1.139 | 8 | 0.916 | 0.989 | 0.073 | 0.917 | 0.072 |
| larvae | red | infected | control | 1.071 | 8 | 0.917 | 0.989 | 0.093 | 0.897 | 0.092 |
| larvae | red | infected | refractory | 1.098 | 8 | 0.869 | 0.983 | 0.082 | 0.902 | 0.081 |
| larvae | red | infected | susceptible | 1.105 | 8 | 0.691 | 0.955 | 0.071 | 0.887 | 0.068 |
| larvae | red | stressed | control | 1.029 | 8 | 0.650 | 0.948 | 0.092 | 0.860 | 0.087 |
| larvae | red | stressed | refractory | 1.021 | 8 | 0.580 | 0.934 | 0.090 | 0.850 | 0.084 |
| larvae | red | stressed | susceptible | 1.103 | 8 | 0.615 | 0.941 | 0.067 | 0.878 | 0.063 |
| larvae | green | uninfected | control | 1.107 | 8 | 0.884 | 0.985 | 0.080 | 0.906 | 0.079 |
| larvae | green | uninfected | refractory | 1.068 | 8 | 0.664 | 0.950 | 0.080 | 0.874 | 0.076 |
| larvae | green | uninfected | susceptible | 1.079 | 8 | 0.732 | 0.962 | 0.081 | 0.884 | 0.078 |
| larvae | green | infected | control | 1.093 | 8 | 0.860 | 0.981 | 0.083 | 0.900 | 0.082 |
| larvae | green | infected | refractory | 1.019 | 8 | 0.896 | 0.986 | 0.111 | 0.877 | 0.110 |
| larvae | green | infected | susceptible | 1.041 | 8 | 0.680 | 0.953 | 0.090 | 0.867 | 0.086 |
| larvae | green | stressed | control | 1.081 | 8 | 0.819 | 0.975 | 0.085 | 0.893 | 0.083 |
| larvae | green | stressed | refractory | 1.053 | 8 | 0.761 | 0.966 | 0.091 | 0.879 | 0.088 |
| larvae | green | stressed | susceptible | 1.089 | 8 | 0.813 | 0.975 | 0.082 | 0.894 | 0.080 |
|  |  |  |  |  |  |  |  |  |  |  |
| stage | group | environment | genotype | λ  (day^-1^) | T_pupae_  (days) | p_pupae_  (p.emerge) | σ_pupae_  (day^-1^) | γ_pupae_ | P_pupae_  (day^-1^) | G_pupae_  (day^-1^) |
| pupae | black | uninfected | control | 1.083 | 2 | 0.900 | 0.949 | 0.467 | 0.506 | 0.443 |
| pupae | black | uninfected | refractory | 1.081 | 2 | 0.900 | 0.949 | 0.467 | 0.505 | 0.443 |
| pupae | black | uninfected | susceptible | 1.091 | 2 | 0.900 | 0.949 | 0.465 | 0.507 | 0.441 |
| pupae | black | infected | control | 1.150 | 2 | 0.900 | 0.949 | 0.452 | 0.520 | 0.429 |
| pupae | black | infected | refractory | 1.114 | 2 | 0.900 | 0.949 | 0.460 | 0.512 | 0.436 |
| pupae | black | infected | susceptible | 1.125 | 2 | 0.900 | 0.949 | 0.458 | 0.515 | 0.434 |
| pupae | black | stressed | control | 1.142 | 2 | 0.900 | 0.949 | 0.454 | 0.518 | 0.430 |
| pupae | black | stressed | refractory | 1.031 | 2 | 0.900 | 0.949 | 0.479 | 0.494 | 0.455 |
| pupae | black | stressed | susceptible | 1.100 | 2 | 0.900 | 0.949 | 0.463 | 0.509 | 0.439 |
| pupae | red | uninfected | control | 1.147 | 2 | 0.900 | 0.949 | 0.453 | 0.519 | 0.430 |
| pupae | red | uninfected | refractory | 1.078 | 2 | 0.900 | 0.949 | 0.468 | 0.505 | 0.444 |
| pupae | red | uninfected | susceptible | 1.139 | 2 | 0.900 | 0.949 | 0.454 | 0.518 | 0.431 |
| pupae | red | infected | control | 1.071 | 2 | 0.900 | 0.949 | 0.470 | 0.503 | 0.446 |
| pupae | red | infected | refractory | 1.098 | 2 | 0.900 | 0.949 | 0.464 | 0.509 | 0.440 |
| pupae | red | infected | susceptible | 1.105 | 2 | 0.900 | 0.949 | 0.462 | 0.510 | 0.438 |
| pupae | red | stressed | control | 1.029 | 2 | 0.900 | 0.949 | 0.480 | 0.494 | 0.455 |
| pupae | red | stressed | refractory | 1.021 | 2 | 0.900 | 0.949 | 0.482 | 0.492 | 0.457 |
| pupae | red | stressed | susceptible | 1.103 | 2 | 0.900 | 0.949 | 0.462 | 0.510 | 0.439 |
| pupae | green | uninfected | control | 1.107 | 2 | 0.900 | 0.949 | 0.462 | 0.511 | 0.438 |
| pupae | green | uninfected | refractory | 1.068 | 2 | 0.900 | 0.949 | 0.471 | 0.502 | 0.446 |
| pupae | green | uninfected | susceptible | 1.079 | 2 | 0.900 | 0.949 | 0.468 | 0.505 | 0.444 |
| pupae | green | infected | control | 1.093 | 2 | 0.900 | 0.949 | 0.465 | 0.508 | 0.441 |
| pupae | green | infected | refractory | 1.019 | 2 | 0.900 | 0.949 | 0.482 | 0.491 | 0.457 |
| pupae | green | infected | susceptible | 1.041 | 2 | 0.900 | 0.949 | 0.477 | 0.496 | 0.452 |
| pupae | green | stressed | control | 1.081 | 2 | 0.900 | 0.949 | 0.467 | 0.505 | 0.443 |
| pupae | green | stressed | refractory | 1.053 | 2 | 0.900 | 0.949 | 0.474 | 0.499 | 0.450 |
| pupae | green | stressed | susceptible | 1.089 | 2 | 0.900 | 0.949 | 0.466 | 0.507 | 0.442 |
|  |  |  |  |  |  |  |  |  |  |  |
| stage | group | environment | genotype | λ  (day^-1^) | T_virgin_  (days) | p_virgin_  (p.mate) | σ_virgin_  (day^-1^) | γ_virgin_ | P_virgin_  (day^-1^) | G_virgin_  (day^-1^) |
| virgins | black | uninfected | control | 1.083 | 2 | 0.900 | 0.949 | 0.467 | 0.506 | 0.443 |
| virgins | black | uninfected | refractory | 1.081 | 2 | 0.900 | 0.949 | 0.467 | 0.505 | 0.443 |
| virgins | black | uninfected | susceptible | 1.091 | 2 | 0.900 | 0.949 | 0.465 | 0.507 | 0.441 |
| virgins | black | infected | control | 1.150 | 2 | 0.900 | 0.949 | 0.452 | 0.520 | 0.429 |
| virgins | black | infected | refractory | 1.114 | 2 | 0.900 | 0.949 | 0.460 | 0.512 | 0.436 |
| virgins | black | infected | susceptible | 1.125 | 2 | 0.900 | 0.949 | 0.458 | 0.515 | 0.434 |
| virgins | black | stressed | control | 1.142 | 2 | 0.900 | 0.949 | 0.454 | 0.518 | 0.430 |
| virgins | black | stressed | refractory | 1.031 | 2 | 0.900 | 0.949 | 0.479 | 0.494 | 0.455 |
| virgins | black | stressed | susceptible | 1.100 | 2 | 0.900 | 0.949 | 0.463 | 0.509 | 0.439 |
| virgins | red | uninfected | control | 1.147 | 2 | 0.900 | 0.949 | 0.453 | 0.519 | 0.430 |
| virgins | red | uninfected | refractory | 1.078 | 2 | 0.900 | 0.949 | 0.468 | 0.505 | 0.444 |
| virgins | red | uninfected | susceptible | 1.139 | 2 | 0.900 | 0.949 | 0.454 | 0.518 | 0.431 |
| virgins | red | infected | control | 1.071 | 2 | 0.900 | 0.949 | 0.470 | 0.503 | 0.446 |
| virgins | red | infected | refractory | 1.098 | 2 | 0.900 | 0.949 | 0.464 | 0.509 | 0.440 |
| virgins | red | infected | susceptible | 1.105 | 2 | 0.900 | 0.949 | 0.462 | 0.510 | 0.438 |
| virgins | red | stressed | control | 1.029 | 2 | 0.900 | 0.949 | 0.480 | 0.494 | 0.455 |
| virgins | red | stressed | refractory | 1.021 | 2 | 0.900 | 0.949 | 0.482 | 0.492 | 0.457 |
| virgins | red | stressed | susceptible | 1.103 | 2 | 0.900 | 0.949 | 0.462 | 0.510 | 0.439 |
| virgins | green | uninfected | control | 1.107 | 2 | 0.900 | 0.949 | 0.462 | 0.511 | 0.438 |
| virgins | green | uninfected | refractory | 1.068 | 2 | 0.900 | 0.949 | 0.471 | 0.502 | 0.446 |
| virgins | green | uninfected | susceptible | 1.079 | 2 | 0.900 | 0.949 | 0.468 | 0.505 | 0.444 |
| virgins | green | infected | control | 1.093 | 2 | 0.900 | 0.949 | 0.465 | 0.508 | 0.441 |
| virgins | green | infected | refractory | 1.019 | 2 | 0.900 | 0.949 | 0.482 | 0.491 | 0.457 |
| virgins | green | infected | susceptible | 1.041 | 2 | 0.900 | 0.949 | 0.477 | 0.496 | 0.452 |
| virgins | green | stressed | control | 1.081 | 2 | 0.900 | 0.949 | 0.467 | 0.505 | 0.443 |
| virgins | green | stressed | refractory | 1.053 | 2 | 0.900 | 0.949 | 0.474 | 0.499 | 0.450 |
| virgins | green | stressed | susceptible | 1.089 | 2 | 0.900 | 0.949 | 0.466 | 0.507 | 0.442 |
|  |  |  |  |  |  |  |  |  |  |  |
| stage | group | environment | genotype | λ  (day^-1^) | T_mated_  (days) | p_mated_  p.blood*  p.surv.blood | σ_mated_  (day^-1^) | γ_mated_ | P_mated_  (day^-1^) | G_mated_  (day^-1^) |
| mated | black | uninfected | control | 1.083 | 2 | 0.613 | 0.783 | 0.420 | 0.454 | 0.328 |
| mated | black | uninfected | refractory | 1.081 | 2 | 0.663 | 0.814 | 0.429 | 0.464 | 0.350 |
| mated | black | uninfected | susceptible | 1.091 | 2 | 0.738 | 0.859 | 0.440 | 0.480 | 0.378 |
| mated | black | infected | control | 1.150 | 2 | 0.897 | 0.947 | 0.452 | 0.519 | 0.428 |
| mated | black | infected | refractory | 1.114 | 2 | 0.768 | 0.876 | 0.440 | 0.491 | 0.386 |
| mated | black | infected | susceptible | 1.125 | 2 | 0.918 | 0.958 | 0.460 | 0.517 | 0.441 |
| mated | black | stressed | control | 1.142 | 2 | 0.696 | 0.834 | 0.422 | 0.482 | 0.352 |
| mated | black | stressed | refractory | 1.031 | 2 | 0.517 | 0.719 | 0.411 | 0.424 | 0.296 |
| mated | black | stressed | susceptible | 1.100 | 2 | 0.669 | 0.818 | 0.426 | 0.469 | 0.349 |
| mated | red | uninfected | control | 1.147 | 2 | 1.000 | 1.000 | 0.466 | 0.534 | 0.466 |
| mated | red | uninfected | refractory | 1.078 | 2 | 0.558 | 0.747 | 0.409 | 0.441 | 0.306 |
| mated | red | uninfected | susceptible | 1.139 | 2 | 0.853 | 0.923 | 0.448 | 0.510 | 0.413 |
| mated | red | infected | control | 1.071 | 2 | 0.722 | 0.850 | 0.442 | 0.474 | 0.376 |
| mated | red | infected | refractory | 1.098 | 2 | 0.700 | 0.837 | 0.433 | 0.475 | 0.362 |
| mated | red | infected | susceptible | 1.105 | 2 | 0.699 | 0.836 | 0.431 | 0.476 | 0.360 |
| mated | red | stressed | control | 1.029 | 2 | 0.563 | 0.750 | 0.422 | 0.434 | 0.316 |
| mated | red | stressed | refractory | 1.021 | 2 | 0.513 | 0.716 | 0.412 | 0.421 | 0.295 |
| mated | red | stressed | susceptible | 1.103 | 2 | 0.850 | 0.922 | 0.455 | 0.502 | 0.420 |
| mated | green | uninfected | control | 1.107 | 2 | 0.745 | 0.863 | 0.438 | 0.485 | 0.378 |
| mated | green | uninfected | refractory | 1.068 | 2 | 0.747 | 0.864 | 0.447 | 0.478 | 0.387 |
| mated | green | uninfected | susceptible | 1.079 | 2 | 0.720 | 0.849 | 0.440 | 0.475 | 0.373 |
| mated | green | infected | control | 1.093 | 2 | 0.660 | 0.813 | 0.427 | 0.466 | 0.347 |
| mated | green | infected | refractory | 1.019 | 2 | 0.491 | 0.700 | 0.407 | 0.415 | 0.285 |
| mated | green | infected | susceptible | 1.041 | 2 | 0.700 | 0.837 | 0.445 | 0.464 | 0.373 |
| mated | green | stressed | control | 1.081 | 2 | 0.650 | 0.807 | 0.427 | 0.462 | 0.345 |
| mated | green | stressed | refractory | 1.053 | 2 | 0.377 | 0.614 | 0.368 | 0.388 | 0.226 |
| mated | green | stressed | susceptible | 1.089 | 2 | 0.709 | 0.842 | 0.436 | 0.475 | 0.367 |
|  |  |  |  |  |  |  |  |  |  |  |
| stage | group | environment | genotype | λ  (day^-1^) | T_gravid_  (days) | p_gravid_ (p.surv.ovip) | σ_gravid_  (day^-1^) | γ_gravid_ | P_gravid_  (day^-1^) | G_gravid_  (day^-1^) |
| gravid | black | uninfected | control | 1.083 | 4 | 0.980 | 0.995 | 0.219 | 0.777 | 0.218 |
| gravid | black | uninfected | refractory | 1.081 | 4 | 0.925 | 0.981 | 0.215 | 0.770 | 0.210 |
| gravid | black | uninfected | susceptible | 1.091 | 4 | 0.932 | 0.983 | 0.212 | 0.774 | 0.209 |
| gravid | black | infected | control | 1.150 | 4 | 0.918 | 0.979 | 0.193 | 0.790 | 0.189 |
| gravid | black | infected | refractory | 1.114 | 4 | 0.905 | 0.975 | 0.203 | 0.778 | 0.198 |
| gravid | black | infected | susceptible | 1.125 | 4 | 0.947 | 0.987 | 0.203 | 0.786 | 0.201 |
| gravid | black | stressed | control | 1.142 | 4 | 0.967 | 0.992 | 0.200 | 0.794 | 0.198 |
| gravid | black | stressed | refractory | 1.031 | 4 | 0.685 | 0.910 | 0.205 | 0.723 | 0.187 |
| gravid | black | stressed | susceptible | 1.100 | 4 | 0.933 | 0.983 | 0.210 | 0.777 | 0.206 |
| gravid | red | uninfected | control | 1.147 | 4 | 0.950 | 0.987 | 0.197 | 0.793 | 0.194 |
| gravid | red | uninfected | refractory | 1.078 | 4 | 0.906 | 0.976 | 0.214 | 0.767 | 0.209 |
| gravid | red | uninfected | susceptible | 1.139 | 4 | 0.961 | 0.990 | 0.200 | 0.792 | 0.198 |
| gravid | red | infected | control | 1.071 | 4 | 0.932 | 0.983 | 0.219 | 0.768 | 0.215 |
| gravid | red | infected | refractory | 1.098 | 4 | 0.946 | 0.986 | 0.211 | 0.778 | 0.209 |
| gravid | red | infected | susceptible | 1.105 | 4 | 0.897 | 0.973 | 0.205 | 0.774 | 0.199 |
| gravid | red | stressed | control | 1.029 | 4 | 0.667 | 0.904 | 0.204 | 0.720 | 0.184 |
| gravid | red | stressed | refractory | 1.021 | 4 | 0.854 | 0.961 | 0.228 | 0.742 | 0.219 |
| gravid | red | stressed | susceptible | 1.103 | 4 | 0.941 | 0.985 | 0.209 | 0.779 | 0.206 |
| gravid | green | uninfected | control | 1.107 | 4 | 0.914 | 0.978 | 0.206 | 0.777 | 0.201 |
| gravid | green | uninfected | refractory | 1.068 | 4 | 0.955 | 0.989 | 0.222 | 0.769 | 0.219 |
| gravid | green | uninfected | susceptible | 1.079 | 4 | 0.972 | 0.993 | 0.220 | 0.775 | 0.218 |
| gravid | green | infected | control | 1.093 | 4 | 0.779 | 0.940 | 0.197 | 0.755 | 0.185 |
| gravid | green | infected | refractory | 1.019 | 4 | 0.696 | 0.914 | 0.211 | 0.721 | 0.192 |
| gravid | green | infected | susceptible | 1.041 | 4 | 0.812 | 0.949 | 0.216 | 0.744 | 0.205 |
| gravid | green | stressed | control | 1.081 | 4 | 0.867 | 0.965 | 0.209 | 0.763 | 0.202 |
| gravid | green | stressed | refractory | 1.053 | 4 | 0.909 | 0.976 | 0.223 | 0.759 | 0.217 |
| gravid | green | stressed | susceptible | 1.089 | 4 | 0.919 | 0.979 | 0.212 | 0.772 | 0.207 |
|  |  |  |  |  |  |  |  |  |  |  |
| stage | group | environment | genotype |  |  | G_gravid_  (day^-1^) | eggs.tot  (eggs) | p.lay | p.fem | F_gravid_ |
| gravid | black | uninfected | control |  |  | 0.218 | 50.5 | 0.553 | 0.5 | 3.041 |
| gravid | black | uninfected | refractory |  |  | 0.210 | 71.8 | 0.516 | 0.5 | 3.894 |
| gravid | black | uninfected | susceptible |  |  | 0.209 | 60.0 | 0.562 | 0.5 | 3.520 |
| gravid | black | infected | control |  |  | 0.189 | 79.0 | 0.821 | 0.5 | 6.129 |
| gravid | black | infected | refractory |  |  | 0.198 | 98.4 | 0.571 | 0.5 | 5.550 |
| gravid | black | infected | susceptible |  |  | 0.201 | 75.4 | 0.768 | 0.5 | 5.810 |
| gravid | black | stressed | control |  |  | 0.198 | 66.1 | 0.871 | 0.5 | 5.702 |
| gravid | black | stressed | refractory |  |  | 0.187 | 42.6 | 0.817 | 0.5 | 3.253 |
| gravid | black | stressed | susceptible |  |  | 0.206 | 76.4 | 0.849 | 0.5 | 6.682 |
| gravid | red | uninfected | control |  |  | 0.194 | 68.1 | 0.662 | 0.5 | 4.388 |
| gravid | red | uninfected | refractory |  |  | 0.209 | 58.2 | 0.693 | 0.5 | 4.207 |
| gravid | red | uninfected | susceptible |  |  | 0.198 | 84.2 | 0.621 | 0.5 | 5.186 |
| gravid | red | infected | control |  |  | 0.215 | 32.7 | 0.656 | 0.5 | 2.308 |
| gravid | red | infected | refractory |  |  | 0.209 | 58.7 | 0.616 | 0.5 | 3.775 |
| gravid | red | infected | susceptible |  |  | 0.199 | 52.7 | 0.706 | 0.5 | 3.708 |
| gravid | red | stressed | control |  |  | 0.184 | 25.8 | 0.782 | 0.5 | 1.854 |
| gravid | red | stressed | refractory |  |  | 0.219 | 34.6 | 0.543 | 0.5 | 2.055 |
| gravid | red | stressed | susceptible |  |  | 0.206 | 48.6 | 0.678 | 0.5 | 3.396 |
| gravid | green | uninfected | control |  |  | 0.201 | 70.7 | 0.661 | 0.5 | 4.699 |
| gravid | green | uninfected | refractory |  |  | 0.219 | 77.1 | 0.519 | 0.5 | 4.390 |
| gravid | green | uninfected | susceptible |  |  | 0.218 | 83.0 | 0.318 | 0.5 | 2.880 |
| gravid | green | infected | control |  |  | 0.185 | 57.6 | 0.676 | 0.5 | 3.600 |
| gravid | green | infected | refractory |  |  | 0.192 | 50.8 | 0.239 | 0.5 | 1.167 |
| gravid | green | infected | susceptible |  |  | 0.205 | 39.4 | 0.396 | 0.5 | 1.603 |
| gravid | green | stressed | control |  |  | 0.202 | 38.4 | 0.694 | 0.5 | 2.688 |
| gravid | green | stressed | refractory |  |  | 0.217 | 50.9 | 0.820 | 0.5 | 4.534 |
| gravid | green | stressed | susceptible |  |  | 0.207 | 46.1 | 0.456 | 0.5 | 2.180 |
